# Minimal prevalence of Huntington’s disease in the South of Brazil and instability of the expanded CAG tract during intergenerational transmissions

**DOI:** 10.1590/1678-4685-GMB-2018-0032

**Published:** 2019-06-27

**Authors:** Raphael Machado de Castilhos, José Augusto dos Santos, Marina Coutinho Augustin, José Luiz Pedroso, Orlando Barsottini, Roberta Saba, Henrique Ballalai Ferraz, Clécio Godeiro, Fernando Regla Vargas, Diego Zanotti Salarini, Gabriel Vasata Furtado, Marcia Polese-Bonatto, Luiza Paulsen Rodrigues, Lucas Schenatto Sena, Maria Luiza Saraiva-Pereira, Laura Bannach Jardim, RedeNeurogenética

**Affiliations:** 1 Programa de Pós-graduação em Genética e Biologia Molecular, Universidade Federal do Rio Grande do Sul (UFRGS), Porto Alegre, Rio Grande do Sul, Brazil; 2 Programa de Pós-graduação em Bioquímica, Universidade Federal do Rio Grande do Sul (UFRGS), Porto Alegre, Rio Grande do Sul, Brazil; 3 Programa de Pós-graduação em Biologia Celular e Molecular, Universidade Federal do Rio Grande do Sul (UFRGS), Porto Alegre, Rio Grande do Sul, Brazil; 4 Departamento de Bioquímica, Universidade Federal do Rio Grande do Sul (UFRGS), Porto Alegre, Rio Grande do Sul, Brazil; 5 Departamento de Medicina Interna, Universidade Federal do Rio Grande do Sul (UFRGS), Porto Alegre, Rio Grande do Sul, Brazil; 6 Faculdade de Medicina, Universidade Federal do Rio Grande do Sul (UFRGS), Porto Alegre, Rio Grande do Sul, Brazil; 7 Disciplina de Neurologia Clínica, Escola Paulista de Medicina, Universidade Federal de São Paulo (UNIFESP), São Paulo, SP, Brazil; 8 Departamento de Medicina Integrada, Universidade Federal do Rio Grande do Norte (UFRN), Natal, RN, Brazil; 9 Hospital Graffrée e Guinle, Universidade Federal do Estado do Rio de Janeiro (UNIRIO), Rio de Janeiro, RJ, Brazil; 10 Laboratório de Epidemiologia de Malformações Congênitas, Instituto Oswaldo Cruz, Fundação Oswaldo Cruz, Rio de Janeiro, RJ, Brazil; 11 Santa Casa de Misericórdia de São Paulo, São Paulo, SP, Brazil; 12 Laboratório de Identificação Genética, Centro de Pesquisa Experimental, Hospital de Clinicas de Porto Alegre (HCPA), Porto Alegre, RS, Brazil; 13 Serviço de Genética Médica, Hospital de Clinicas de Porto Alegre (HCPA), Porto Alegre, RS, Brazil; 14 Rede Neurogenética, Centro de Pesquisa Clínica, Hospital de Clínicas de Porto Alegre (HCPA), Porto Alegre, RS, Brazil; 15 Instituto Nacional de Genética Médica Populacional (INAGEMP), Porto Alegre, RS, Brazil

**Keywords:** CAG expansion, Huntington’s disease, intergenerational instability, minimal prevalence

## Abstract

Huntington’s disease (HD) is due to dominant expansions of the CAG repeat of the *HTT* gene. Meiotic instability of the (CAG)_n_ might impact the disorder frequency. We report on HD minimal prevalence in Rio Grande do Sul (RS) state, Brazil, and on intergenerational instability of the (CAG)_n_ in HD families. Symptomatic and at-risk subjects from 179 HD families were ascertained between 2013 and 2016. Clinical, molecular and family history data were obtained. Expanded (CAG)_n_ length differences between parent and child (delta-expanded-(CAG)_n_) were calculated. Effect of parental age on the (CAG)_n_ instability upon transmission was inferred by correlating delta-expanded-(CAG)_n_ between siblings to their age differences. HD minimal prevalence in RS state was estimated as 1.85:100,000 inhabitants. Alleles with (CAG)_27-35_ were found on 21/384 non-disease associated chromosomes (5.5%); among 253 expanded alleles, four (1.6%) were within reduced penetrance range with (CAG)_36-39_. In 32 direct transmissions, mean instability was larger among paternal than maternal transmissions. In direct transmissions and in 51 sibling pairs, parental age at the time of child birth were not correlated with delta-expanded-(CAG)_n_. Briefly, HD prevalence in RS state was lower than those reported for European populations. Expanded (CAG)_n_ transmissions were unstable and not associated to parental age.

## Introduction

Huntington’s disease (HD) is an autosomal dominant neurodegenerative disorder characterized by chorea and other motor impairments, behavioral disturbances, and dementia. HD is caused by expansion of the CAG repeat in the *HTT* gene to 36 or more trinucleotides, leading to an abnormally long polyglutamine (polyQ) tract in the encoded protein huntingtin (Hoogeveen *et al.*, 1993; The Huntington’s disease Collaborative Research Group, 1993). As in other polyQ diseases, an inverse correlation between expanded (CAG)_n_ length and age at onset (AO) is observed. The expanded allele is unstable upon cellular divisions including gametogenesis, which brings variability to phenotypic expression and penetrance (Andrew *et al.*, 1993; Duyao *et al.*, 1993).

Instabilities of the expanded (CAG)_n_ in meiosis might explain, at least partially, some clinical phenomena associated with HD: further expansions are associated to anticipation, while contractions in past generations might be related to non-penetrance and lack of family history (Telenius *et al.*, 1994; Sequeiros *et al.*, 2010). Male transmissions are more unstable than female transmissions of expanded (CAG)_n_; in fact, expansions occur more frequently than contractions when the father is the transmitting genitor, in cohorts reported so far. Reasons for this remain unclear (Wheeler *et al.*, 2007; Aziz *et al.*, 2011). Considering that our timeframe as researchers favors pairing more severe offspring with less severe parents and not the opposite, the potential effect of an observation bias cannot be completely ruled out (Maciel *et al.*, 1995; Souza *et al.*, 2016). This bias is boosted if offspring recruitment is not balanced, including preferentially symptomatic children.

Studies on intergenerational transmissions of expanded (CAG)_n_ included predominantly North American and European HD families (MacDonald *et al.*, 1993; Zuhlke *et al.*, 1993; Telenius *et al.*, 1994; Kremer *et al.*, 1995; Wheeler *et al.*, 2007; Ramos *et al.*, 2012). In contrast, the most studied HD group has a Latin American origin, this being the HD cohort of Maracaibo, Venezuela. Since this group probably shares a common ancestor (Ávila-Girón, 1973), its characteristics may not be generalizable to HD individuals with other origins. In any case, controversial findings on the effect of age of the affected parent on the transmission of expanded CAG repeats were obtained in the Maracaibo cohort (Leeflang *et al.*, 1999; Wheeler *et al.*, 2007).

Knowledge about HD in all other Latin American populations and families is very scarce. For instance, prevalence rates are lacking in most regions of the continent. Therefore, our aim was to estimate the minimal prevalence of HD in Brazil and to investigate if intergenerational instability of the expanded CAG repeat differed from that documented by studies in other populations, pointing to population stratification related to environmental and/or genetic factors.

## Material and Methods

Symptomatic HD individuals with molecular diagnosis performed in our institution, as well as their caregivers, were contacted from September 2013 to December 2016. Part of this cohort has been reported elsewhere, in the context of differential diagnosis of Huntington’s disease (Castilhos *et al.*, 2014). Those individuals and their relatives at 50% and 25% risk of developing HD were invited to participate in this study. At risk relatives who had not been tested previously were informed and agreed not to receive laboratory results obtained in the present study. Those who decided to undergo pre-symptomatic testing (PST) were referred to the PST program of our institution. After consent, age at onset (AO) of the symptomatic individuals and age, gender, gender of the transmitting parent, as well as age of the parent at childbirth were obtained for all participants. Complete pedigrees with updated information about living and deceased individuals were built. Blood samples were collected and analyzed at our institution, as previously described (Castilhos *et al.*, 2014). The results of molecular testing of asymptomatic individuals were added to the database by the PI of this study (LBJ), who did not contact directly the participants under study. Subsequently, data were made anonymous before the analyses to guarantee confidentiality of all subjects and families.

Stability of expanded (CAG)_n_ upon transmissions was inferred by the difference between the (CAG)_n_ length of parent and child: delta-expanded-(CAG)_n_. A stable CAG transmission was defined by delta-expanded-(CAG)_n_ equal to zero. Cases of intergenerational expansion were defined by delta-expanded-(CAG)_n_ equal to or greater than 1 CAG trinucleotide, while contractions were defined by delta-expanded-(CAG)_n_ equal to or lesser than -1 CAG trinucleotide.

In kindreds where information on (CAG)_n_ expansion from the transmitting parent was not available, the effect of age of the parent on the instability of (CAG)_n_ transmission was estimated by correlating the delta-expanded-(CAG)_n_ between siblings to their age differences, assuming the older sibling as carrying the reference expanded (CAG)_n_ for a given kindred.

Variables AO, delta AO, expanded (CAG)_n_ and delta-expanded-(CAG)_n_ were described as medians and range. Non-parametric statistics were used, as most variables did not have normal distributions, and *p*=0.05 was chosen as the significant level. Data were computed into electronic files and pedigrees were built in Progeny© software. All data were analyzed in the Statistical Package for the Social Sciences (SPSS) v 18.

Informed consent was obtained from each participant. This study was approved by the Ethics Committee (EC) from the Hospital de Clínicas de Porto Alegre (GPPG number 13-0182) and by ECs from the other participanting institutions (Hospital São Paulo - UNIFESP and Hospital Graffrée e Guinle -UNIRIO). This study was registered at Plataforma Brasil as CAAE 15493313.2.0000.5327.

## Results

In total, 179 Brazilian families were ascertained. From those, 99 were from Rio Grande do Sul state. Prevalence was estimated for this state, as data obtained for this state were broader than those obtained in other parts of the country. Families from Rio Grande do Sul included 209 symptomatic HD patients, 690 individuals at 50% risk, and 515 at 25% risk individuals, alive by December 2016. Distribution of HD families was proportional to population dispersion in this state (Figure S1). Considering the estimated population in 2017, based in the most recent (2010) demographic census (Instituto Brasileiro de Geografia e Estatística - IBGE, 2017), the minimal prevalence of symptomatic HD patients and of at 50% risk individuals were 1.85 and 6:100,000 inhabitants, respectively. The remaining 80 HD families (110 individuals) were from other regions of Brazil, where minimal prevalence was not estimated.

### CAG repeats in Brazilian families

Molecular data were obtained from 253 carriers of expanded alleles (213 symptomatic and 40 asymptomatic subjects; including one symptomatic subject with expanded repeats in both alleles), and 66 non-carriers (controls) from the 179 Brazilian families. In summary, there were 253 expanded and 384 non-disease associated alleles.

**Figure 1:**
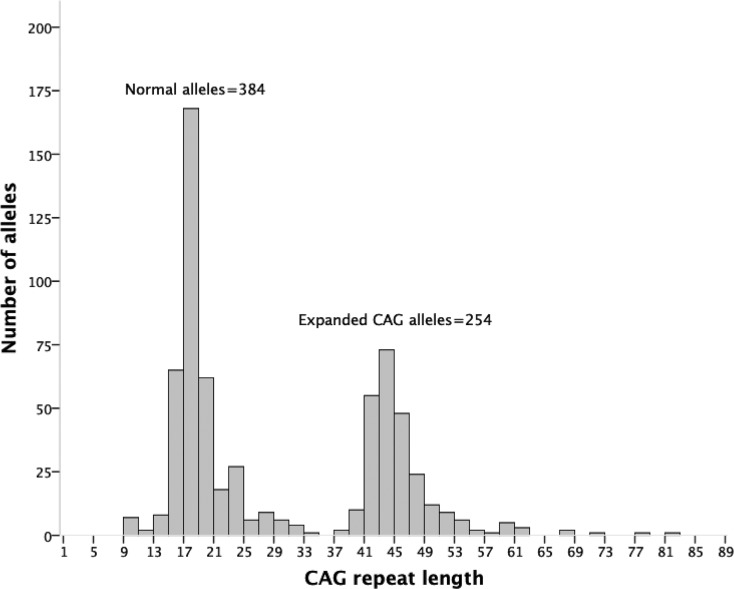
Distribution of normal and expanded CAG repeat lengths in carriers and non-carriers of expanded alleles.

The distribution of non-disease associated alleles did not differ from a normal distribution, with a CAG repeat mean ± sd (range) of 18 ± 3.4 (9 to 33 trinucleotides). Intermediate alleles with (CAG)_27-35_ were found on 21/384 non-disease associated chromosomes (5.5%) (Table 1 and Figure 1).

**Table 1:** CAG repeat length, allele categories, age at onset and age of participants in the study.

		HD carriers			Non-carriers	Total
		Total	Symptomatic carriers	Asymptomatic carriers		
Number of subjects		253	213	40	66	319
Expanded CAG repeat -median (range)		44 (37-81)	44 (39-81) ^a^	43 (37-48) ^a^	-	
Normal CAG repeat - median (range)		17 (9-33)	17 (10-33)	17 (9-37)	17 (10-31)	
Number of alleles	Stable (CAG)_£_26	240 ^b^	203 ^b^	37	123	363
	Intermediate (CAG)_27-35_	12	9	3	9	21
	Reduced penetrance (CAG)_36-39_	4	2	2	-	4
	Full-penetrant (CAG)_40 or more_	250 ^b^	212 ^b^	38	-	250
Age at disease onset (years)		-	39 ± 12 (range 6-67)	-	-	
Age (years) (mean ± SD)		-	50.5 ± 11 (range 18-73) ^c^	35.85 ± 12 (range 19-77)	40 ± 13.75 (range 19-76)	

a *p*< 0.05, Mann-Whitney U-test;

b one individual had a full penetrant expanded CAG repeat in both alleles;

c *p*< 0.001, one-way ANOVA (Tukey)

Among the 253 expanded alleles, four (1.6%) were within the reduced penetrance range with (CAG)_36-39_, while 249 (98.4%) were fully penetrant alleles with (CAG)_40 or more_ (Table 1 and Figure 1). Two carriers of reduced penetrant alleles remained asymptomatic at 45 and 39 years of age. Both were offspring of carriers of penetrant alleles: an individual with a (CAG)_39_ allele was the child of a (CAG)_42_ allele carrier, the other, with a (CAG)_37_ allele, was the child of a (CAG)_43_ allele carrier. CAG expansion, AO and age of the participants are shown in Table 1. As reported previously (Castilhos *et al.*, 2014), AO was inversely correlated with CAG expansion, with rho = - 0.7 (*p*<0.0001, Spearman).

Interestingly, we identified a female carrier with an expanded (CAG)_40_ allele who was still asymptomatic at 77 years of age. Her five children were all asymptomatic; none of them agreed to participate in the present study. Her 69 year-old brother carried a (CAG)_43_ allele and was symptomatic since his forties.

### Instability of expanded CAG repeats upon transmissions

Thirty-two direct transmissions (13 paternal and 19 maternal) were documented in the cohort, with 28/32 children still asymptomatic. These data are presented in Table 2. Mean ± sd instability was 1.37 ± 5.85 in the whole sample. Instability upon paternal transmissions (3.77 ± 8.22) was larger and more related to further expansions than upon maternal transmissions (-0.26 ± 2.64) and the difference remained significant when only asymptomatic children were included (Mann-Whitney: *p*=0.006).

**Table 2:** Intergenerational transmissions of expanded *HTT* CAG repeat, according to gender of affected parent.

	Total	Paternal	Maternal	*p*
n	32	13	19	
Asymptomatic children	28/32	11/13	17/19	ns (Chi-squared)
Delta-expanded (CAG)_n_ Mean (SD)	1.37 (5.85)	3.77 (8.22)	- 0.26 (2.64)	0.005 (Mann-Whitney)
(CAG)_n_ upon transmission:				
Expanded	11 (34.4%)	9 (69.2%)	2 (10.5%	0.004 (Fisher)
Stable	14 (43.75%)	3 (23%)	11(57.9%)	
Contracted	7 (21.8%)	1 (7.7%)	6 (31.6%)	
Parental Expanded (CAG)_n_ Median (range)	43 (39-60)	43 (41-51)	44 (39-60)	ns (Mann-Whitney)
Age (yrs) of affected parent at child birth (mean ± SD)	26 ± 6	27.7 ± 6	24.8 ± 6	0.020 (Mann-Whitney)

**Figure 2:**
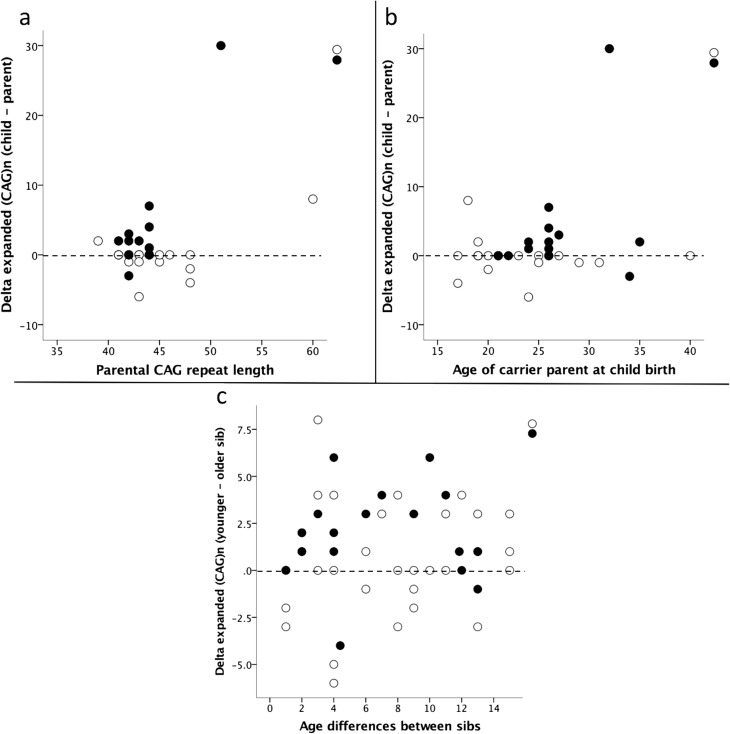
Instability of expanded CAG repeats upon transmissions (dashed line corresponds to stability of the CAG repeat transmission). (a) Delta-expanded-(CAG)n child-parent according to parental CAG repeat length; (b) Delta-expanded-(CAG)n child-parent according to parental age at child-birth; (c) Delta-expanded-(CAG)n between siblings according to their age differences and gender of affected parent. Full circles: paternal transmissions; empty circles: maternal transmissions.

Parental CAG repeat expansion and age at the time of child birth were not correlated with delta-expanded-(CAG)_n_, both in the whole sample as well as according to parental gender (Figure 2A,B). No significant differences were detected in expanded CAG transmissions to male and female offspring, both in the whole sample and when parental gender was considered (Mann-Whitney; data not shown).

Fifty-one pairs of siblings were studied, with 40 (78.4%) of them showing discordant expanded CAG repeat lengths. Larger CAG repeat in the younger sib was more frequent upon paternal (14/19;73.7%) than maternal transmission (13/30; 43.3%) (Chi-squared: *p*=0.037), although overall discordant lengths of expanded CAG repeats between sibs were similar upon paternal (16/19; 84.2%) and maternal transmissions (22/30; 73.3%) (Chi-squared). Initially, a unidirectional effect was investigated, according to the hypothesis that the older the parent, the larger would be further expansions in the offspring. In paternal transmissions, most of the expanded CAG repeats of the younger sib (11 out of 16) were larger than those of the oldest sib. In spite of this, delta-expanded-(CAG)_n_ and age differences between siblings were not correlated in the overall group or in male and female transmissions (Spearman) (Figure 2C). Thereafter, a bidirectional effect was tested, i.e., the effect of aging on instability, irrespective of this being due to further expansion or contraction. Again, no correlation was found between delta-expanded-(CAG)_n_ and age of parent at child-birth, even when analyzing paternal and maternal transmissions separately (Spearman; data nor shown).

## Discussion

The minimal prevalence of HD in Rio Grande do Sul, Brazil, was estimated to be 1.85/100,000. Dispersion of non-disease associated alleles and proportions of intermediate and reduced penetrant alleles were similar to those found in European studies. Forty four percent of expanded CAG repeat transmissions to symptomatic and asymptomatic offspring were stable, the remainder being contracted or expanded. As reported before, expanded were more common than stable or contracted CAG repeats upon paternal transmission. Neither the CAG expansion nor the age of the parent at the time of child birth were significant modifiers of expanded CAG repeat instability upon transmission, but the small number of transmissions especially from male carriers might have reduced the power of this observation.

HD due to expansions of the CAG repeat of the *HTT* gene is by far the most common diagnosis in families with HD phenotype in Brazil (Castilhos *et al.*, 2014). However, population frequencies were not obtained before this present survey. HD suspicion is usually straightforward, and our institution is the only one to perform molecular analysis of *HTT* for the public health system in the South of Brazil. The estimated prevalence of 1.85/100,000 inhabitants in Rio Grande do Sul is minimal, but should not be too different than the actual one. For instance, we would need to detect another 355 hypothetical patients (or 71 families, based on our ratio of 2.1 symptomatic individuals per family) to achieve a prevalence similar to those obtained in European populations, this being 5/100,000 (Rawlins *et al.*, 2016). We do not believe that so many undetected cases would be living in this region. Interestingly, our minimum prevalence was more similar to that found in the Venezuelan population (except Maracaibo), which is 0.5/100,000 inhabitants (Paradisi *et al.*, 2008), than to that obtained in European countries. Since the haplotype background from HD families from Rio Grande do Sul was quite the same as that from European families (Castilhos *et al.*, 2016), we believe that most of these HD families are related to European carriers settled at our country. The low prevalence would be related to the subsequent ethnic mixture, since not only Europeans but also Amerindian and African ancestors contributed to the present population of Brazil (Wang *et al.*, 2008).

Intermediate alleles were found in 5.5% of all non-disease associated alleles, while proportions obtained in European populations varied between 3 and 5.8% (Sequeiros *et al.*, 2010; Semaka *et al.*, 2013). Reduced penetrant alleles corresponded to 1.6% of all expanded alleles of the present Brazilian cohort, compared to rates obtained in other populations that ranged from 1.6 to 7.7% (Wexler *et al.*, 2004; Sequeiros *et al.*, 2010; Dorsey *et al.*, 2013). Whether our data are due to a relatively small sampling, or whether they reflect real frequencies of intermediate and reduced penetrant alleles in Brazil, remains to be established.

Rates of expansions and contractions upon expanded CAG repeat transmissions in our cohort were similar to those obtained in other populations (MacDonald *et al.*, 1993; Zuhlke *et al.*, 1993; Telenius *et al.*, 1994; Wheeler *et al.*, 2007). Considering that most of the CAG repeat expansions were detected upon transmissions from asymptomatic subjects, there is no reason to suspect of important observational biases in our study. Moreover, although the number of asymptomatic carriers of expanded CAG repeats – some of them related to documented contractions – were small, the chance that their offspring grows unaware of HD is real. Further recurrences of symptomatic carriers in their progeny might be interpreted as isolated cases, in the future, complicating genetic counseling and preventive measures. The relatively frequent contractions upon transmissions of expanded CAG repeats (21.8%) should make counselors aware of this phenomenon.

Expansions and contractions occurred in both maternal, as well as in paternal transmissions, as in previous studies (Wheeler *et al.*, 2007; Aziz *et al.*, 2011; Ramos *et al.*, 2012). Although instability of expanded CAG repeats was more frequent in paternal transmissions, 42.1% of maternal transmissions were also unstable. Thus, mechanisms favoring the instability of the expanded CAG repeats operate in both lineages, probably in oogenesis and spermatogenesis. Instabilities in maternal CAG repeat transmissions might be related to DNA repair errors related to the protracted meiosis (Pearson *et al.*, 2005; Mirkin 2007). In paternal transmissions, the multiple cellular divisions associated to spermatogenesis are usually evoked to explain the expanded repeat instability. In the Maracaibo cohort, Leeflang *et al.* (1999) showed that the range and mean allele length increased with age, according to data obtained from sperm cells analysis of 27 HD carriers. In the same cohort, “a weak, non-significant effect of the age of the transmitting men on repeat-length change (*p*=0.05, Bonferroni-corrected *p*=0.26)” was detected in 83 male transmissions (Wheeler *et al.*, 2007). Two other studies were unable to find any correlation between changes in the transmitted expanded repeat and parental age at childbirth, but they did not split male from female transmissions (Aziz *et al.*, 2011; Ramos *et al.*, 2012).

In the time interval of our study, we obtained a considerable number of parent and child pairs. Getting DNA samples from affected parents and children, both alive, is quite difficult in such a severe disease, especially during only a three-year period of collection. We have tried to increase sample size using another strategy to study the relationship between the delta-expanded-(CAG)_n_ and delta of age from sib pairs, as previously done in other polyglutamine diseases (Souza *et al.*, 2016). This indirect way to test transmission increased the number of observations to 51 pairs of sibs. Since most of these observations included at least one asymptomatic sib, the risk of bias may have been small. Though most male transmissions were related to further expansions in the second sib, the observation did not achieve significance.

Finally, the effect of parental expanded CAG repeat length on transmission instability seen in other cohorts (Wheeler *et al.*, 2007; Aziz *et al.*, 2011; Ramos *et al.*, 2012) was not observed in our population. This might be due to the small number of transmissions in this study.

In conclusion, we have demonstrated that HD prevalence in Rio Grande do Sul, Brazil was lower than that observed in predominantly European populations. Expanded CAG repeat transmissions were very unstable in both paternal and maternal lineages, and frequent occurrences of further expansions in paternal and contractions in maternal transmissions were confirmed. Although the age of the affected father at childbirth was not correlated with the aggravation of expanded CAG repeat instability, this result might be due to a type II error and deserves further investigation.

## Supplementary material

The following online material is available for this article:

Figure S1 Origin of HD families from Rio Grande do Sul, compared with absolute numbers of inhabitants.
