# Supplementary material for: Minimal prevalence of Huntington’s disease in the South of Brazil and instability of the expanded CAG tract during intergenerational transmissions
Source: Genet Mol Biol. 2019 Jun 27;42(2):329–36. doi: 10.1590/1678-4685-GMB-2018-0032 (PMC6726154; doi:10.1590/1678-4685-GMB-2018-0032)
Supplement: Supplementary file 1 [file 1415-4757-GMB-1678-4685-GMB-2018-0032-20190515-suppl.pdf]

Supplementary Material to "Minimal prevalence of Huntington's disease in the South of Brazil and instability of the expanded CAG tract during intergenerational transmissions"

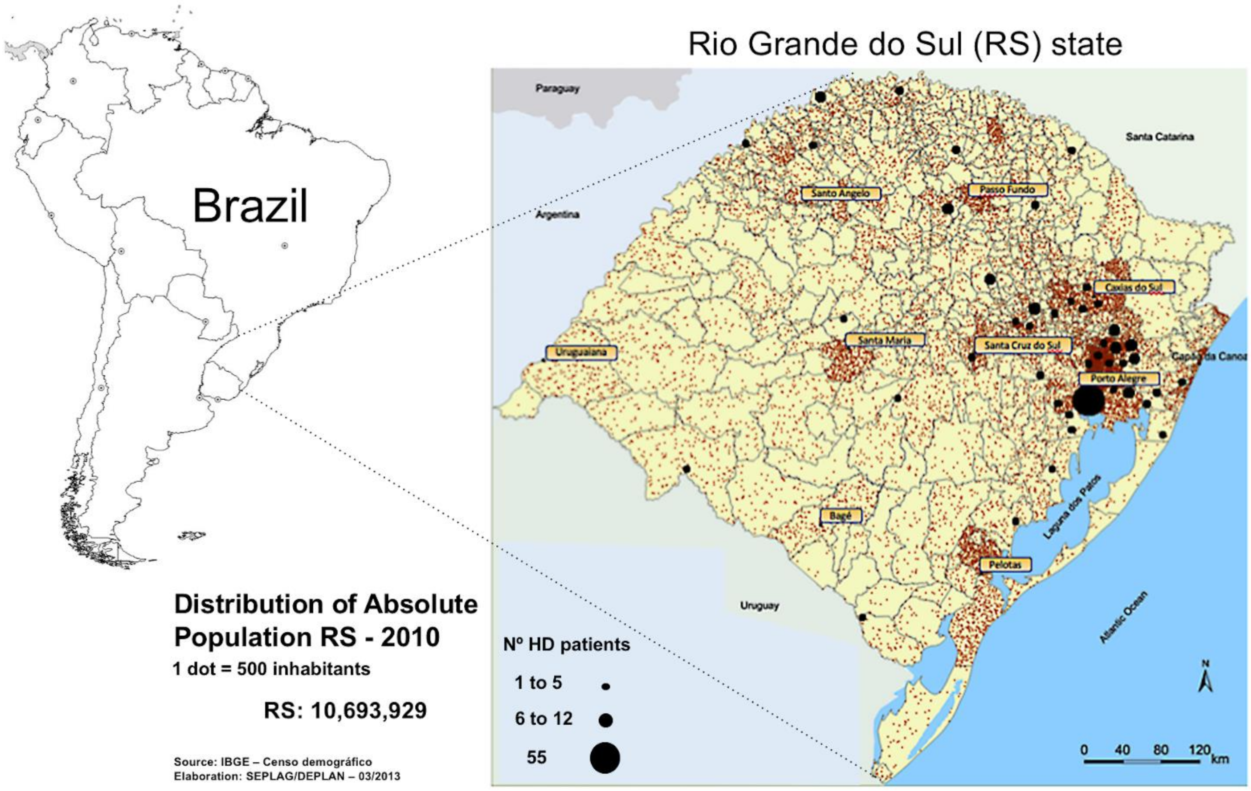

Figure S1 - Origin of HD families from Rio Grande do Sul, compared with absolute numbers of inhabitants.
